# Differential expression of metabotropic glutamate and GABA receptors at neocortical glutamatergic and GABAergic axon terminals

**DOI:** 10.3389/fncel.2015.00345

**Published:** 2015-09-04

**Authors:** Luca Bragina, Tiziana Bonifacino, Silvia Bassi, Marco Milanese, Giambattista Bonanno, Fiorenzo Conti

**Affiliations:** ^1^Section of Neuroscience and Cell Biology, Department of Experimental and Clinical Medicine, Università Politecnica delle MarcheAncona, Italy; ^2^Center for Neurobiology of Aging, Istituto Nazionale di Riposo e Cura per Anziani – Istituto di Ricovero e Cura a Carattere ScientificoAncona, Italy; ^3^Department of Pharmacy, Unit of Pharmacology and Toxicology, University of GenoaGenoa, Italy; ^4^Center of Excellence for Biomedical Research, University of GenoaGenoa, Italy; ^5^Fondazione di Medicina Molecolare, Università Politecnica delle MarcheAncona, Italy

**Keywords:** glutamate, GABA, metabotropic receptors, heteroreceptors, autoreceptors, cerebral cortex

## Abstract

Metabotropic glutamate (Glu) receptors (mGluRs) and GABA_B_ receptors are highly expressed at presynaptic sites. To verify the possibility that the two classes of metabotropic receptors contribute to axon terminals heterogeneity, we studied the localization of mGluR1α, mGluR5, mGluR2/3, mGluR7, and GABA_B1_ in VGLUT1-, VGLUT2-, and VGAT- positive terminals in the cerebral cortex of adult rats. VGLUT1-positive puncta expressed mGluR1α (∼5%), mGluR5 (∼6%), mGluR2/3 (∼22%), mGluR7 (∼17%), and GABA_B1_ (∼40%); VGLUT2-positive terminals expressed mGluR1α (∼10%), mGluR5 (∼11%), mGluR2/3 (∼20%), mGluR7 (∼28%), and GABA_B1_ (∼25%); whereas VGAT-positive puncta expressed mGluR1α (∼27%), mGluR5 (∼24%), mGluR2/3 (∼38%), mGluR7 (∼31%), and GABA_B1_ (∼19%). Control experiments ruled out the possibility that postsynaptic mGluRs and GABA_B1_ might have significantly biased our results. We also performed functional assays in synaptosomal preparations, and showed that all agonists modify Glu and GABA levels, which return to baseline upon exposure to antagonists. Overall, these findings indicate that mGluR1α, mGluR5, mGluR2/3, mGluR7, and GABA_B1_ expression differ significantly between glutamatergic and GABAergic axon terminals, and that the robust expression of heteroreceptors may contribute to the homeostatic regulation of the balance between excitation and inhibition.

## Introduction

Presynaptic mechanisms affecting neurotransmitter release play a key role in modulating synaptic strength and plasticity (Atwood and Karunanithi, 2002); they may be associated to molecular heterogeneity of axon terminals (Staple et al., 2000). Following the demonstration that more than 2,000 genes are differentially expressed in glutamatergic and GABAergic neurons (Sugino et al., 2006), we investigated whether glutamatergic and GABAergic release machineries could be differentiated on the basis of the proteins they express, and showed that in cerebral cortex the expression pattern of several presynaptic proteins involved in transmitter release (including synapsins, synaptophysins, synaptosomal-associated proteins, synaptogyrins, synaptobrevin/vesicle-associated membrane proteins, syntaxins, synaptotagmins, synaptic vesicle proteins, and Rab3) varies both between glutamatergic and GABAergic terminals and between VGLUT1+ and VGLUT2+ glutamatergic terminals (Bragina et al., 2007, 2010, 2011).

Metabotropic glutamate (Glu) receptors (mGluRs) and GABA_B_ modulates neuronal excitability, transmitter release, and synaptic plasticity (Bowery et al., 1980; Herrero et al., 1992; Pin and Duvoisin, 1995). mGluRs consist of at least eight subtypes classified into three groups: group I receptors (mGluR1 and 5), coupled to the Gq signal pathway, are predominantly postsynaptic, and generally potentiate neuronal excitability; group II (mGluR2 and 3) and group III receptors (mGluR 4, 6, 7, and 8), linked to Gi/Go and thus cAMP inhibitors, are predominantly presynaptic and usually decrease excitability (Shigemoto et al., 1997; Gereau and Swanson, 2008). Notwithstanding the value of this general scheme, it is worth noting that the localization of mGluRs is more complex. Indeed, besides mGluR2/3 and mGluR7, mGluR1α and mGluR5 are also expressed presynaptically at asymmetric and symmetric synapses in different brain regions, including cerebral cortex (Romano et al., 1995; Bradley et al., 1996; Petralia et al., 1996; Lujan et al., 1997; Shigemoto et al., 1997; Kinoshita et al., 1998; Tamaru et al., 2001; Dalezios et al., 2002; Muly et al., 2003; Paquet and Smith, 2003; Somogyi et al., 2003; Rodrigues et al., 2005; Musante et al., 2008; Giribaldi et al., 2013), where they also modulate positively or negatively glutamate and GABA release (Herrero et al., 1992; Battaglia et al., 1997; Li et al., 2008; Musante et al., 2008; Summa et al., 2013).

GABA_B_ are coupled to Gi/Go proteins and decrease neuronal excitability in both pre- and postsynaptic elements; they exist as heterodimers (of GABA_B1_ and GABA_B2_ subunits) which yield functional receptors (Kaupmann et al., 1998; White et al., 1998; Gonchar et al., 2001; Lopez-Bendito et al., 2002; Tabata et al., 2004). GABA_B1_ is expressed presynaptically in several brain regions (Kulik et al., 2002, 2003; Lujan et al., 2004; McDonald et al., 2004; Lacey et al., 2005; Lujan and Shigemoto, 2006), including the cerebral cortex, where it is localized at both symmetric and asymmetric synapses (Gonchar et al., 2001; Lopez-Bendito et al., 2002). One major function of presynaptic GABA_B_ receptors is modulation of neurotransmitter release, as they inhibit the release of both glutamate and GABA in different regions of the CNS, including cerebral cortex (Bowery et al., 1980; Bonanno and Raiteri, 1993).

The widespread localization of mGluRs and GABA_B_ at presynaptic sites raises the possibility that both classes of metabotropic receptors contribute to axon terminals heterogeneity. To verify this hypothesis, we studied the localization of mGluR1α, mGluR5, mGluR2/3, mGluR7, and GABA_B1_ in VGLUT1+, VGLUT2+, and VGAT+^1^ terminals in the cerebral cortex of adult rats. We report that the expression of these receptors differs significantly between glutamatergic and GABAergic axon terminals, and that expression of heteroreceptors is greater than expected. We also performed functional assays in synaptosomal preparations, and showed that all agonists modify Glu and GABA levels, which return to baseline upon antagonists administration.

## Materials and Methods

### Animals and Tissue Preparation

Adult male Sprague–Dawley rats (190–220 g; Charles River, Milan, Italy) were used. All experiments were carried out in accordance with the European Community Council Directive dated November 24, 1986 (86/609 EEC), and were approved by the local authority veterinary service (CESA, Comitato Etico per la Sperimentazione Animale; Università Politecnica delle Marche). Animals were kept under a dark–light cycle of 12 h and permitted food and water *ad libitum.*

For synaptosome purification, release experiments, and western blotting, rats were euthanized and brains rapidly removed. Homogenization of neocortex, membrane preparation, protein determination, SDS-PAGE analysis, and immunoblotting were performed as described (Bragina et al., 2006). Precast gels (Tris-HCl; BioRad, Hercules, CA, USA) were used at 4–20% polyacrylamide concentration for metabotropic receptors (15 μg total proteins) and at 7.5% for the vesicular transporters (10 μg total proteins).

For immunocytochemical studies, rats were anesthetized with chloral hydrate (300 mg/kg i.p.) and perfused through the ascending aorta with saline followed by 4% paraformaldehyde in 0.1 M phosphate buffer (PB; pH 7.4). Brains were post-fixed for 1 day at 4°C in the same fixative, cut with a Vibratome into 30 μm thick sections, and processed.

### Antibodies

The primary antibodies used are listed in **Table 1**. Western blots were performed to verify antibodies specificity; nitrocellulose filters were probed with antibodies to VGLUT1, VGLUT2, VGAT, mGluR1α, mGluR5, mGluR2/3, mGluR7, and GABA_B1_ at the dilutions reported in **Table 1**. After exposure to the appropriate peroxidase-conjugated antibodies (Vector; Burlingame, CA, USA), immunoreactive bands were visualized by BioRad Chemidoc and Quantity One software (BioRad, Hemel Hempstead, UK) using the SuperSignal West Pico (Rockford, IL, USA) chemiluminescent substrate.

**Table 1:** Primary antibodies.

	Host°	Dilution^∗^	Source	Characterization
VGAT	M	1:50 (IF) 1:500 (WB)	Synaptic System/131011	Bogen et al. (2006), Tafoya et al. (2006)
VGLUT1	GP	1:1000 (IF) 1:2000 (WB)	Millipore/AB5905	Melone et al. (2005)
VGLUT2	GP	1:1000 (IF) 1:2000 (WB)	Millipore/AB5907	Cubelos et al. (2005), Liu et al. (2005)
mGluR1α	R	1:250 (IF and WB)	Millipore/AB1551	Muly et al. (2003)
mGluR5	R	1:250 (IF and WB)	Millipore/AB5675	Muly et al. (2003)
mGluR2/3	R	1:50 (IF) 1:250 (WB)	Millipore/AB1553	Petralia et al. (1996)
mGluR7	R	1:250 (IF and WB)	Millipore/07-239	Pinheiro et al. (2007)
GABA_B1_	R	1:250 (IF and WB)	Dr. A. Kulik, Freiburg (Germany)	Kulik et al. (2002)

*°GP, guinea pig; M, mouse; R, rabbit; ^∗^IF, immunofluorescence; WB, western blotting.*

### Co-localization Studies

Sections were incubated for 1 h in normal goat serum (NGS; 10% in PB with 0.2% Triton X-100), and then overnight at room temperature in a solution containing a mixture of the primary antibodies. The next day, sections were incubated in 10% NGS (30 min), and then for 90 min in a mixture of the appropriate secondary fluorescent antibodies. For the VGLUT1 and VGLUT2 series, we used fluorescein isothiocyanate-conjugated goat anti-guinea-pig IgG (FI-7000, Vector; Burlingame, CA, USA) for the vesicular transporters (1:100) and tetramethylrhodamine isothiocyanate-conjugated goat anti-rabbit IgG (T-2769, Molecular Probes; Poort Gebouw, The Netherlands) for the metabotropic receptors (1:100); for the VGAT series, we used fluorescein goat anti-mouse IgG (F-2761, Molecular probes) for the vesicular transporter (1:100) and tetramethylrhodamine isothiocyanate-conjugated goat anti-rabbit IgG (T-2769, Molecular Probes; Poort Gebouw, The Netherlands) for the metabotropic receptors (1:100). Sections were then mounted, air-dried, and coverslipped using Vectashield mounting medium (H-1000; Vector). Double-labeled sections were examined using a Leica (TCS SP2) confocal laser microscope equipped with an argon (488 nm) and a helium/neon (543 nm) laser for excitation of fluorescein isothiocyanate (FITC) and tetramethylrhodamine isothiocyanate (TRITC), respectively. Green and red immunofluorescence were imaged sequentially and emissions separated with 515/30 nm band pass (FITC) and 570 nm long pass (TRITC) filters. Control experiments with single-labeled sections and sections incubated either with two primary and one secondary antibody, or with one primary and two secondary antibodies revealed no appreciable FITC/TRITC bleed-through or antibody cross-reactivity.

Images of *experimental series* were collected from a region of the parietal cortex characterized by a conspicuous layer IV, with intermingled dysgranular regions, densely packed layers II and III, and a relatively cell-free layer Va. This area corresponds to the first somatic sensory cortex (SI), as identified also by Woolsey and LeMessurier (1948), Welker (1971), Zilles et al. (1980), Donoghue and Wise (1982). Images were acquired from randomly selected subfields in layers II–VI (at least 4–6/layer; 2–4 sections/animal; 10 rats). Layer I was not sampled because it hardly contains VGAT+ puncta (Chaudhry et al., 1998; Minelli et al., 2003). Images were acquired using a 63 × oil immersion lens (numerical aperture 1.4; pinhole 1.0 and image size 1,024 × 1,024 pixels, yielding a pixel size of 0.06 μm) from a plane in which the resolution of both stains was optimal and always between 1.3 and 1.8 μm from the surface. To improve the signal/noise ratio, 10 frames/image were averaged.

Quantitative analysis was performed in ∼8,000 randomly selected subfields measuring about 25 × 25 μm from the 1,024 × 1,024 pixel images. In order to minimize the fusion of puncta, the contrast of each image was adjusted manually within the maximum range of levels for each color channel. Analysis of contrast adjustment (not shown) showed that gain/contrast changes within the spectrum used did not alter significantly the percentage of puncta. Then, without reducing the image resolution, the images were binarized and processed by watershed filter using ImageJ software (bfd). Next, each channel was examined separately to identify and count with ImageJ immunopositive puncta; the two channels were then merged and the number of co-localizing puncta was counted manually. Puncta were considered double-labeled when overlap was virtually complete or when a given punctum was entirely included in the other. Moreover, we analyzed ∼2,000 randomly selected subfield (25 × 25 μm) from the 1,024 × 1,024 pixel images acquired in molecular layer of cerebellum and ventrobasal nucleus (10–20/section; 2–4section/animal; 2 animals).

In addition, we compared our manual method with a computerized overlap analysis that defines two objects as co-localized if the centre of mass of one falls within the area of the other (Lachmanovich et al., 2003). To this end, we analyzed about half of all double-labeled sections studied here with the overlap method included in JACoP toolbox of ImageJ (Bolte and Cordelieres, 2006), and found that the percentage of co-localization obtained with the two methods were comparable.

### Synaptosomes Purification

Synaptosomes were prepared from rat neocortex with a protocol modified by Dunkley et al. (1986) and Stigliani et al. (2006). Briefly, rats were sacrificed and brain were rapidly removed. Parietal cortices were homogenized in 10 volume of Tris buffer (4°C; pH 7.4) containing 0.32 M sucrose, EDTA 1 mM and protease inhibitors (Complete EDTA-free; Roche Molecular Biochemicals, Indianapolis, IN, USA), and centrifuged at 1,000 *g* for 5 min to remove nuclei and cellular debris. Subsequently, supernatant was centrifuged at 9,200 *g* for 10 min. Synaptosomal fraction were purified by centrifugation a 33,000 *g* using Percoll-sucrose density gradient (2–6–10–20%) for 5 min. The synaptosomal fraction (10–20%) Percol interface was washed by centrifugation at 20,000 *g* for 15 min at 4°C, and resuspended in fresh physiologic medium having the following composition (in mM): 140 NaCl, 3 KCl, 1.2 MgSO_4_, 1.2 NaH_2_PO_4_, 5 NaHCO_3_, 1.2 CaCl_2_; 10 Hepes, and 10 glucose (pH 7.4) for release experiments.

### Release Experiments

Synaptosomes (from 32 rats) were incubated at 37°C for 15 min; aliquots of synaptosomal suspension (150 μg) were layered on microporous filters placed at the bottom of a set of parallel superfusion chambers maintained at 37°C (Superfusion System; Ugo Basile, Comerio, Italy; (Raiteri et al., 1984). Superfusion was started with standard medium at a rate of 0.5 ml/min and continued for 48 min. In the experiments aimed at measuring basal Glu and GABA release, after 36 min of superfusion to equilibrate the system, fractions were collected according to the following scheme: four 3-min samples (*t* = 36–39, basal release; *t* = 39–42, *t* = 42–45, and *t* = 45–48, drug-induced release). The mGluR1 and mGluR5 agonist 3,5-DHPG (30 μM) was introduced at *t* = 39, after the first sample was collected. When appropriate, the selective mGluR1 and mGluR5 antagonists LY367385 (1 μM) and MPEP (1 μM), respectively, were introduced at *t* = 30 and maintained until the end of the experiment. Drug effects were evaluated by comparing the amount of endogenous Glu or GABA (pmol/mg synaptosomal protein) in the fourth sample collected (in which maximum effect of 3,5-DHPG was generally reached) with the content in the first sample (basal eﬄux). In experiments aimed at measuring the stimulus-evoked Glu and GABA release, after 36 min of superfusion to equilibrate the system, fractions were collected according to the following scheme: two 3-min samples (*t* = 36–39 min and *t* = 45–48 min; basal outflow) before and after one 6-min sample (*t* = 39–45 min; stimulus-evoked release). A 90-s period of stimulation was applied at *t* = 39 min, after the collection of the first sample. Stimulation of synaptosomes was performed by 15 mM KCl, substituting for equimolar concentration of NaCl. The mGluR2 and mGluR3 agonist LY379268 (100 nM), the mGluR7 agonist AMN082 (100 nM) or the GABA_B_ receptor agonist (-)baclofen (10 μM) was introduced at *t* = 39 min concomitantly to the KCl pulse. When appropriate, the selective mGluR2 and mGluR3 antagonist LY341495 (100 nM), the selective mGluR7 antagonist MMPIP (10 nM) or the selective GABA_B_ antagonist CGP52432 (1 μM) was introduced at *t* = 30 min and maintained until the end of the experiment. The stimulus-evoked overflow was estimated by subtracting the endogenous neurotransmitter release content of the two 3-min fractions (basal outflow) from the endogenous neurotransmitter amount collected in the 6-min fraction (stimulus-evoked overflow).

Endogenous Glu and GABA content in the samples collected was measured by high performance liquid chromatography following pre-column derivatization with *o*-phthalaldehyde and gradient separation on a C18 reverse-phase chromatographic column (10 mm × 4.6 mm, 3 μm; at 30°C; Chrompack, Middleburg, The Netherlands) coupled with fluorometric detection (excitation wavelength 350 nm; emission wavelength 450 nm). Homoserine was used as an internal standard (Bonanno et al., 2005). The following buffers were used: solvent A, 0.1 M sodium acetate (pH 5.8)/methanol 80:20; solvent B, 0.1 M sodium acetate (pH 5.8)/methanol, 20:80; solvent C, sodium acetate (pH 6.0)/methanol, 80:20. The gradient program was as follows: 100% C for 4 min from the initiation of the program; 90% A and 10% B in 1 min; 42% A and 58% B in 14 min; 100% B in 1 min; isocratic flow 2 min; 100% C in 3 min; flow rate 0.9 mL/min. Amino acid release content was expressed as picomoles per milligram of synaptosomal protein.

All chemicals were of the purest analytical grade. (s)-3,5-Dihydroxyphenylglycine ((s)3,5-DHPG, ab120007), 2-Methyl-6-(phenylethynyl)pyridine hydrochloride (MPEP hydrochloride, ab120008), (*S*)-(+)-α-Amino-4-carboxy-2-methylbenzeneacetic acid (LY367385, ab120067), (1*R*,4*R*,5*S*,6*R*)-4-amino-2-oxabicyclo[3.1.0]hexane-4,6-dicarboxylic acid (LY379268, ab120196), (2*S*)-2-Amino-2-[(1*S*,2*S*)-2-carboxycycloprop-1-yl]-3-(xanth-9-yl)propanoic acid (LY341495, ab120199), *N*,*N*’-Dibenzhydrylethane-1,2-diamine dihydrochloride (AMN082 dihydrochloride, ab120011), 6-(4-Methoxyphenyl)-5-methyl-3-(4-pyridinyl)isoxazolo[4,5-*c*]pyridin-4(5*H*)-one (MMPIP, ab120245) were from ABCAM (Cambridge, UK).(*R*)-4-Amino-3-(4-chlorophenyl)butanoic acid ((-)baclofen, code 0796) and 3-[[(3,4-Dichlorophenyl)methyl]amino]propyl] diethoxymethyl)phosphinic acid (CGP 52432, code 1246) were from Tocris Bioscience (Bristol, UK).

### Statistical Analysis

Statistical significance was evaluated by non-parametric Mann–Whitney *U* test (for confocal microscopy in cerebellum and ventrobasal complex), and non-parametric one way ANOVA (Kruskal–Wallis with Dunn’s post-test for confocal microscopy and release experiments in cerebral cortex).

## Results

**FIGURE 1:**
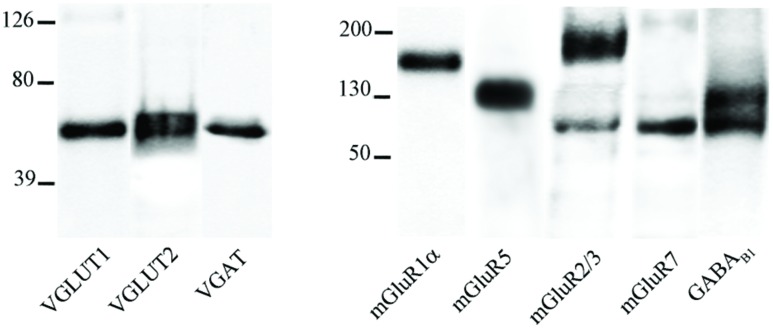
**VGLUT1, VGLUT2, VGAT, mGluR1α, mGluR5, mGluR2/3, mGluR7, and GABA_B1_ antibodies recognized bands of ∼55, 60, 57, 140, 132, 100, and 190, 97, 100, and 132 kDa in the order in crude membrane fractions of rat cerebral cortex**.

In cortical crude membrane fractions, all antibodies recognized bands of the predicted molecular mass (**Figure 1**; Reid et al., 1995; Petralia et al., 1996; Bellocchio et al., 1998; Chaudhry et al., 1998; Kinoshita et al., 1998; Kulik et al., 2002; Varoqui et al., 2002). VGLUT1, VGLUT2, mGluR1α, mGluR5, mGluR2/3, mGluR7, and GABA_B1_ immunoreactivities were as described in previous studies (Reid et al., 1995; Romano et al., 1995; Petralia et al., 1996; Bellocchio et al., 1998; Chaudhry et al., 1998; Kinoshita et al., 1998; Kaneko et al., 2002; Kulik et al., 2002; Lopez-Bendito et al., 2002; Minelli et al., 2003; Muly et al., 2003; Alonso-Nanclares et al., 2004; Conti et al., 2005). These antibodies were, therefore, used to verify whether mGluR1α, mGluR5, mGluR2/3, mGluR7, and GABA_B1_ are differentially expressed in VGLUT1+, VGLUT2+, and VGAT+ axon terminals.

**FIGURE 2:**
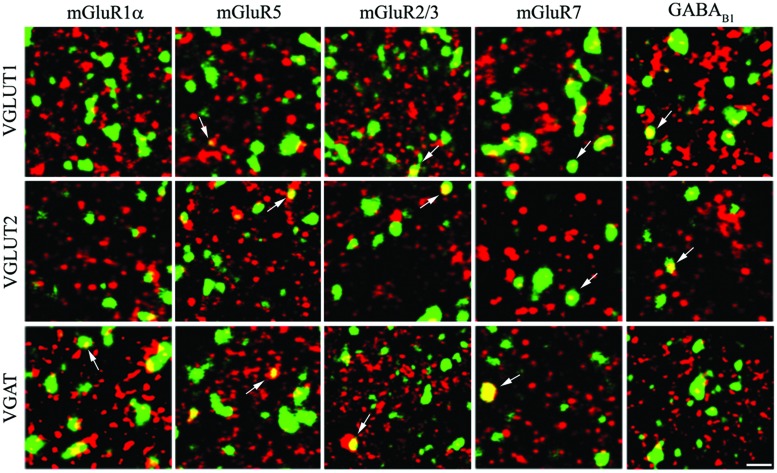
**Expression of metabotropic glutamate and GABA receptors in cerebral cortex.** The figure shows representative images from the VGLUT1 series (first row), the VGLUT2 series (second row), and the VGAT series (third row). Puncta were considered double-labeled (arrows) when overlap was virtually complete or when a given punctum was entirely included in the other puncta. Bar: 4 μm.

**FIGURE 3:**
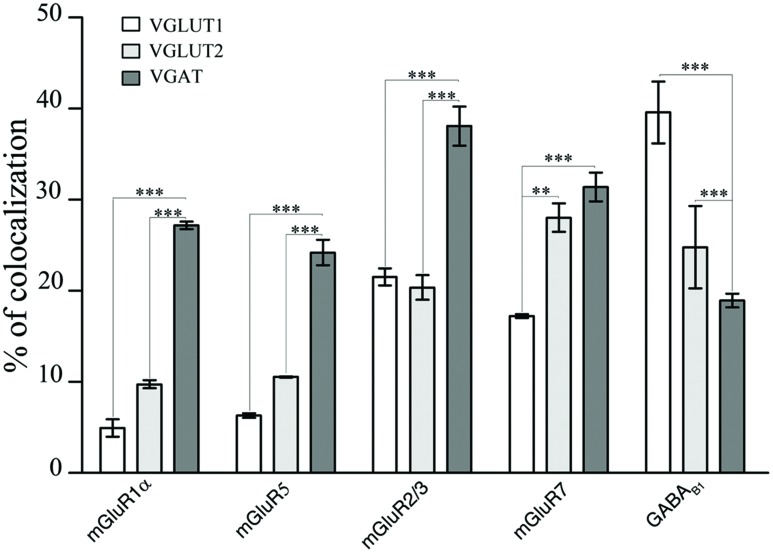
**Expression of mGluR1α, mGluR5, mGluR2/3, mGluR7, and GABA_B1_ in VGLUT1+, VGLUT2+, and VGAT+ axon terminals in cerebral cortex.** Values are mean ± SEM; ^∗∗^*p* < 0.01, ^∗∗∗^*p* < 0.001.

Expression of mGluRs and GABA_B1_ in VGLUT1+ cortical axon terminals was studied in 25 sections from eight rats (**Figures 2** and **3**).

The results showed that 4.93% of VGLUT1+ puncta expressed mGluR1α, 6.31% mGluR5, 22.01% mGluR2/3, 17.23% mGluR7, and 39.59% GABA_B1_ (**Figure 3**; **Table 2**). Analysis of VGLUT2+ cortical axon terminals (30 sections from 10 animals) showed that 9.74% of VGLUT2+ puncta co-localized mGluR1α, 10.55% mGluR5, 20.37% mGluR2/3, 28.03% mGluR7, and 24.79% GABA_B1_ (**Figure 3**; **Table 2**). Analogous studies of VGAT+ puncta (24 sections from 8 rats) revealed that 27.18% of VGAT expressed mGluR1α, 24.20% mGluR5, 38.09% mGluR2/3, 31.39% mGluR7, and 18.94% GABA_B1_ (**Figure 3**; **Table 2**). None of the proteins studied exhibited a significant differential laminar distribution.

**Table 2:** mGluR1α, mGluR5, mGluR2/3, mGluR7, and GABA_B1_ in VGLUT1, VGLUT2, and VGAT puncta.

VT	Puncta (#)	Co-localization	MR
VGLUT1	3841	4.93 ± 0.95%	mGluR1α
	5058	6.31 ± 0.26%	mGluR5
	3276	22.01 ± 0.95%	mGluR2/3
	3925	17.23 ± 0.22%	mGluR7
	3927	39.59 ± 3.39%	GABA_B1_
VGLUT2	3235	9.74 ± 0.43%	mGluR1α
	4444	10.55 ± 0.06%	mGluR5
	6424	20.37 ± 1.36%	mGluR2/3
	3925	28.04 ± 1.57%	mGluR7
	3363	24.79 ± 4.54%	GABA_B1_
VGAT	2942	27.18 ± 0.41%	mGluR1α
	2675	24.2 ± 1.40%	mGluR5
	2732	38.09 ± 2.13%	mGluR2/3
	2615	31.39 ± 1.60%	mGluR7
	2245	18.94 ± 0.75%	GABA_B1_

*VT, vesicular transporter; MR, metabotropic receptor.*

**FIGURE 4:**
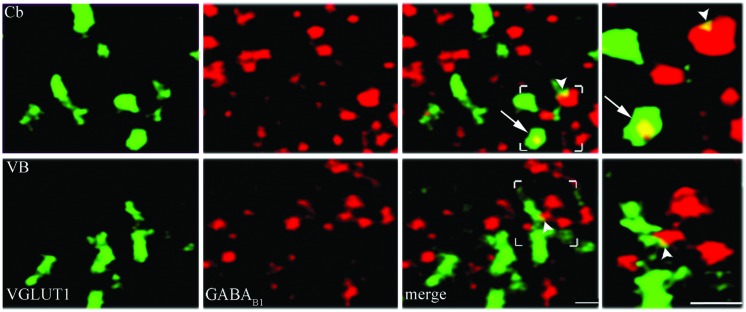
**Control analysis of confocal microscopy images.** VGLUT1/GABA_B1_ in molecular layer of cerebellum (Cb) and in ventrobasal complex (VB). Puncta were considered double-labeled when overlap was virtually complete or when a given punctum was entirely included in the other puncta (arrows in third and fourth columns). Puncta that did not meet these criteria (arrowheads) were not considered double-labeled; Bars: 2 μm.

Since mGluRs and GABA_B_ are located both pre- and postsynaptically, we tested whether our data on pre-synaptic localization had been biased by the presence of postsynaptic receptors. GABA_B1_ is expressed presynaptically in cerebellum but not in the thalamic ventrobasal complex (Kulik et al., 2002); we therefore studied GABA_B1_ expression in VGLUT1, VGLUT2, and VGAT+ puncta in cerebellum and ventrobasal complex (2 rats, 12 sections) to verify whether our confocal method was able to differentiate pre-synaptic from postsynaptic localization. We found that whereas in cerebellum 19 ± 1% of VGLUT1, 9.6 ± 0.3% of VGLUT2, and 9.2 ± 0.3% of VGAT were GABA_B1_+, in ventrobasal complex only 4.9 ± 0.1%, of VGLUT1, 4.4 ± 0.1% of VGLUT2, and 4.2 ± 0.2% of VGAT were GABA_B1_+ (**Figure 4**). These findings indicate that most of labeling identified as presynaptic is indeed presynaptic, even though values given in the preceding paragraph may be slightly overestimated (see Discussion).

**FIGURE 5:**
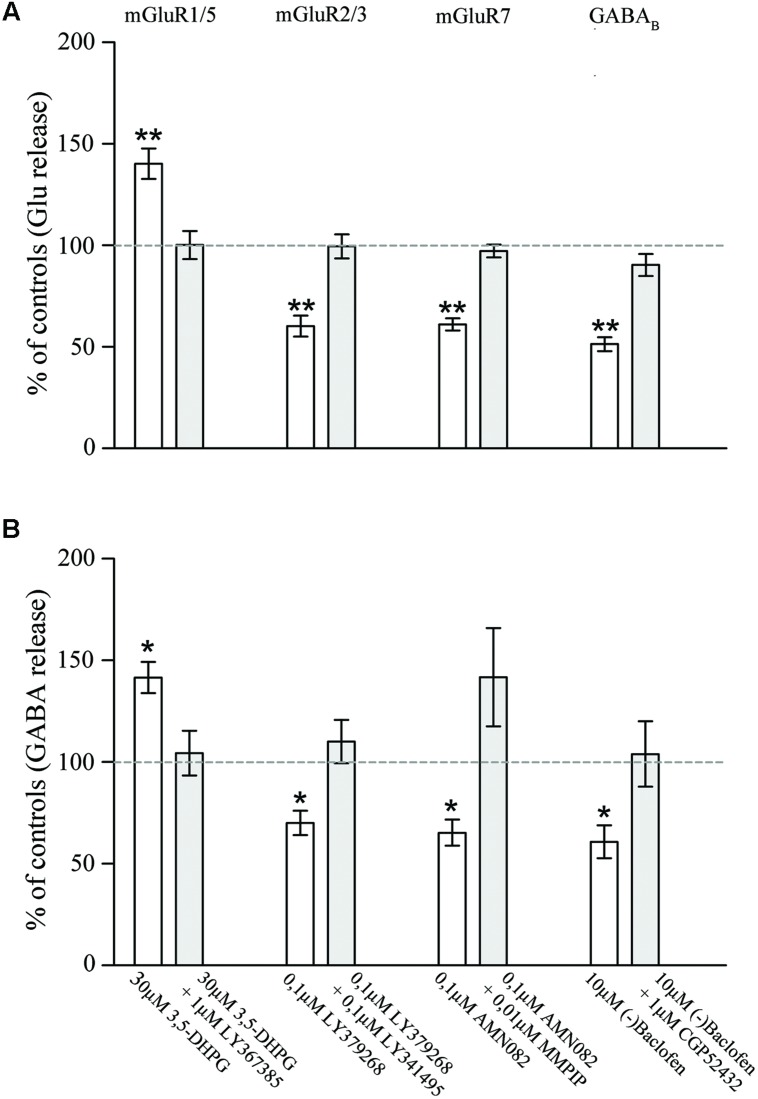
**Effect of agonists and antagonists of mGluR1/5, mGluR2/3, mGluR7, or GABA_B_ receptors on the release of endogenous glutamate **(A)** and GABA **(B)** from rat parietal cortex synaptosomes.** Data are expressed as percent of the spontaneous outflow (mGluR1/5 effect) or of the 15 mM KCl-evoked overflow (mGluR2/3/7 and GABA_B_ effects). White and gray columns refer to agonist or agonist + antagonist administration. Data are means ± SEM of 6–10 independent experiments (6–10 rats per group). ^∗^*p* < 0.05 and ^∗∗^*p* < 0.01 vs. controls.

To further confirm the presynaptic localization of mGluRs and GABA_B1_ and to gain insights on their functional role, we studied the impact of activation of the two classes of metabotropic receptors on the release of endogenous glutamate and GABA in rat parietal cortex. The spontaneous eﬄux of GABA and Glu from cerebral cortex synaptosomes in superfusion, in the absence of stimulus amounted to 51.4 ± 4.62 and to 99.6 ± 4.95 pmol/mg of proteins, respectively. The 15 mM KCl-evoked GABA and Glu overflow amounted to 215.83 ± 17 and to 482.15 ± 38.2 pmol/mg of proteins, respectively. 3,5-DHPG (30 μM), an agonist of mGluR1/5 (Wisniewski and Car, 2002), increased the spontaneous outflow of Glu from rat parietal cortex synaptosomes in superfusion by 40.26 ± 7.45%; its effect was counteracted by the mGluR1 antagonist LY367385 (1 μM; Bruno et al., 1999) and the mGluR5 antagonist MPEP (1 μM; Gasparini et al., 1999; **Figure 5**). Similar results were obtained by measuring the potentiation by DHPG (41.60 ± 7.65%) and the antagonism by LY367385 and MPEP on the basal release of GABA (**Figure 5**). LY379268 (0.1 μM), a selective mGluR2/3 agonist (Monn et al., 1999), significantly reduced the 15 mM KCl-evoked overflow of Glu (by 40.23 ± 5.2%; **Figure 5**) and of GABA (by 30 ± 6%; **Figure 5**); both effects were reverted by 0.01 μM LY341495, a selective mGluR2/3 antagonist (Kingston et al., 1998). AMN082 (0.1 μM), a selective mGluR7 agonist (Mitsukawa et al., 2005), inhibited the release of Glu (by 39.08 ± 3.07%; (**Figure 5**) and GABA (35.3 ± 6.45%; (**Figure 5**) induced by 15 mM KCl; both effects were reverted by 0.01 μM MMPIP, a selective mGluR7 antagonist (Suzuki et al., 2007). Next, we analyzed the effect of (-)baclofen, a selective GABA_B_ receptor agonist (Bowery et al., 1980), on the release of glutamate and GABA induced by 15 mM KCl. **Figure 5** shows that the release of glutamate induced by depolarization was significantly reduced (by 49.37 ± 3.39%) by 10 μM(-)baclofen. The effect of (-)baclofen was reverted by the selective GABA_B_ receptor antagonist CGP52432 (1 μM) (Froestl et al., 1995). (-)baclofen (10 μM) also inhibited the 15 mM KCl-evoked release of GABA (reducing it by 40.79 ± 8.12%), and its effect was counteracted by 1 μM CGP52432 (**Figure 5**). These studies confirm and extend the results of previous studies, by showing that presynaptic mGluRs modulate and GABA_B_ reduces Glu and GABA release and that the effects on the release of the two amino acid were comparable.

## Discussion

The present study showed that mGluRs and GABA_B_ receptors are differentially expressed in glutamatergic and GABAergic axon terminals. Interestingly, mGluR1α, mGluR5, mGluR2/3, and mGluR7 are more robustly expressed at GABAergic than at glutamatergic terminals, whereas GABA_B1_ is more expressed in glutamatergic terminals. In addition, we showed that in cerebral cortex mGluR1 and mGluR5 potentiate Glu and GABA release, whereas mGluR2/3, mGluR7, and GABA_B_ receptors inhibit it.

As reported in Introduction, the mGluRs studied in the present investigation have a presynaptic localization. Indeed, besides mGluR2/3 – a paradigmatic presynaptic mGluR (Ohishi et al., 1994; Neki et al., 1996; Petralia et al., 1996; Lujan et al., 1997; Tamaru et al., 2001), presynaptic expression of mGluR1α, mGluR5 (which are mostly postsynaptic) and mGluR7 in cerebral cortex has been documented (Romano et al., 1995; Bradley et al., 1996; Shigemoto et al., 1996, 1997; Kinzie et al., 1997; Kinoshita et al., 1998; Kosinski et al., 1999; Dalezios et al., 2002; Muly et al., 2003; Somogyi et al., 2003). However, some of the mGluRs studied and GABA_B_ receptors are expressed both pre- and postsynaptically (Kulik et al., 2002; Lopez-Bendito et al., 2002; Boyes and Bolam, 2003; Kulik et al., 2003; Chen et al., 2004; Lujan et al., 2004; Lacey et al., 2005; Lujan and Shigemoto, 2006). Therefore, we performed several control studies to rule out the possibility that our “presynaptic” population had been contaminated by postsynaptic receptors. Since presynaptic localization of GABA_B1_ occurs in cerebellum but not in the thalamic ventrobasal (VB) nucleus (Kulik et al., 2002), we first studied the co-localization of GABA_B1_ receptor and VGLUT1, VGLUT2, and VGAT in both cerebellum and VB, and found a significant differences between co-localization in cerebellum and VB (where co-localization was very low) in the three types of terminals. It is therefore safe to assume that our confocal method can satisfactorily discriminate presynaptic and postsynaptic expression. This view is further reinforced by the observation that in our experimental conditions GABA_B1_ agonists elicited modulatory responses. Next, we performed functional assays in synaptosomal preparations, which provided further evidence that the mGluRs and GABA_B_ studied immunocytochemically are indeed located presynaptically. To this purpose, we monitored Glu and GABA release in cerebral cortex synaptosomes under superfusion conditions (Raiteri et al., 1984). It has been demonstrated that by this way all the released transmitters/modulators are immediately removed by the superfusion medium flow, before they can interact with receptors or other proteins expressed at the presynaptic level. As a consequence, any drug-induced effect on the release of a given neurotransmitter can be attributed exclusively to the direct action of the drug at the nerve terminal storing and releasing that neurotransmitter. Thus, the modulation of Glu or GABA release reported here is in all likelihood due to activation of mGluRs or GABA_B1_ receptors located at Glu or GABA releasing nerve terminals, respectively. Overall, these sets of data provide evidence that, although we might have slightly (<5%) overestimated the population of mGluRs and GABA_B_ receptors, our immunocytochemical localization of presynaptic receptors was not grossly biased by their postsynaptic localization. The present observation that in adult neocortex mGlu and GABA_B1_ receptors exhibit differences between glutamatergic and GABAergic terminals expands the notion that these axon terminals greatly differ in their complement of presynaptic proteins (Bragina et al., 2007, 2010, 2011).

The present results show that not all glutamatergic terminals express mGluRs. Indeed, our data indicate that at least 60% of VGLUT1+ and >10% of VGLUT2+ glutamatergic terminals are devoid of glutamate-mediated metabotropic control. Moreover, these percentages could be significantly increased by the possibility that some terminals express more than one mGluR. As far as VGAT+ terminals are concerned, the present data suggest that all of them could express mGluRs, although the lack of triple-labeling studies prevent a firm conclusion, and that only a small fraction do exhibit GABA-mediated metabotropic control. Thus, although the amount of data available is still limited, it is safe to conclude that not all glutamatergic and GABAergic terminals are controlled through metabotropic receptors by glutamate and GABA, respectively.

Besides this, our results provide some novel data on mGluRs and GABA_B_ receptors localization in cerebral cortex. mGluRs and GABA_B_ receptors have been described in both glutamatergic and GABAergic terminals (Gereau and Swanson, 2008; Raiteri, 2008, for reviews), but the present quantitative observation in neocortex that presynaptic mGluRs are so strongly expressed in GABAergic and that presynaptic GABA_B_ receptors are more expressed in glutamatergic than in GABAergic terminals adds much to previous observations and opens a new perspective to unravel cortical microcircuits. Indeed, although the complex relationships between glutamatergic and GABAergic are only beginning to be unveiled (Lujan et al., 2005; Pinheiro and Mulle, 2008; Heja et al., 2009), making any hypothesis fragile, it is conceivable that the strong heterolocalization of mGluRs and GABA_B_ receptors at cortical axon terminals is part of the complex homeostatic mechanism regulating the balance between excitation and inhibition.

## Conflict of Interest Statement

The authors declare that the research was conducted in the absence of any commercial or financial relationships that could be construed as a potential conflict of interest.

## Footnotes

1 The vesicular transporter described by McIntire et al. (1997) and Sagné et al. (1997) does transport both GABA and glycine (Wojcik et al., 2006), and is termed either VGAT (McIntire et al., 1997) or VIAAT (Sagné et al., 1997). Here, in line with its widespread usage and with the more prominent role of GABA in neocortex, we follow the terminology of McIntire et al. (1997).
